# Seasonal variability of vitamin D status in patients with inflammatory bowel disease – A retrospective cohort study

**DOI:** 10.1371/journal.pone.0217238

**Published:** 2019-05-23

**Authors:** Christine Elisabeth Janssen, Anna Maria Globig, Andrea Busse Grawitz, Dominik Bettinger, Peter Hasselblatt

**Affiliations:** 1 Department of Medicine II, Medical Center–University of Freiburg, Faculty of Medicine, University of Freiburg, Germany; 2 Berta-Ottenstein-Programme, Faculty of Medicine, University of Freiburg, Germany; 3 Institute for Clinical Chemistry and Laboratory Medicine, Medical Center—University of Freiburg, Faculty of Medicine, University of Freiburg, Germany; Medical University of Gdańsk, POLAND

## Abstract

**Objectives:**

Vitamin D deficiency predicts unfavorable disease outcomes in inflammatory bowel disease. Endogenous vitamin D synthesis is affected by seasonal factors including sunlight exposure, raising the question whether seasonality determines the risk of vitamin D deficiency and may mask other clinical risk factors.

**Methods:**

Univariable and multiple regression analyses were performed in a retrospective cohort of 384 patients to determine risk factors for vitamin D deficiency. Since the observed 25-hydroxyvitamin D [25(OH)D] concentrations followed a sinusoidal pattern over the year, all 25(OH)D concentrations were normalized for the predicted variability of the respective day of analysis based on a sinusoidal regression analysis of 25(OH)D test results obtained in more than 86,000 control serum samples.

**Results:**

Vitamin D deficiency was highly prevalent in patients with Crohn’s disease or ulcerative colitis (63% and 55%, respectively) and associated with winter/spring seasons. After normalization of 25(OH)D concentrations for the day of analysis, vitamin D deficiency was associated with histories of complications related to inflammatory bowel disease, surgery, smoking and ongoing diarrhea while initial disease manifestation during adulthood, ongoing vitamin D supplementation and diagnosis of ulcerative colitis *vs*. Crohn’s disease appeared to be protective. Multiple regression analyses revealed that vitamin D deficiency was associated with disease activity in Crohn’s disease and anemia in ulcerative colitis patients. Only few deficient patients achieved sufficient 25(OH)D concentrations over time. However, increasing 25(OH)D concentrations correlated with improved Crohn’s disease activity.

**Conclusions:**

Vitamin D deficiency was highly prevalent in patients with Crohn’s disease and ulcerative colitis and dependent on the season of the year. Following normalization for seasonality by sinusoidal regression analysis, vitamin D deficiency was found to be associated with parameters of complicated disease course while increasing 25(OH)D concentrations over time correlated with reduced activity of Crohn’s disease.

## Introduction

The fat-soluble vitamin D is absorbed in the small intestine or endogenously synthesized in the skin in response to ultraviolet B ray (UVB) exposure. Vitamin D is hydroxylated in the liver to yield 25-hydroxvitamin D [25(OH)D], the major circulating form and most important indicator of the body’s vitamin D stores. 25(OH)D is further hydroxylated in the kidney and other organs including immune cells, resulting in its biologically active form 1,25-dihydroxyvitamin D [1,25(OH)_2_D)], which binds to the vitamin D receptor and thereby controls gene transcription in several organs. Besides its established functions on calcium homeostasis and bone metabolism, vitamin D has been implicated in the pathogenesis of inflammatory bowel disease (IBD, i.e. Crohn’s disease [CD] and ulcerative colitis [UC])[1]. IBD result from uncontrolled inflammatory responses towards intestinal microbiota in patients with genetic predisposition and exposure to environmental factors [2]. Vitamin D was shown to modulate the composition of the intestinal microbiota in CD patients, affect intestinal barrier functions and promote anti-inflammatory signaling in innate and adaptive immune cells [1, 3, 4]. In keeping with this notion, healthy women with predicted vitamin D deficiency are at increased risk to develop CD, but not UC [5]. However, another recent prospective study was unable to demonstrate any correlation between vitamin D intake as well as 25(OH)D serum concentrations and incidence of CD or UC [6]. Vitamin D deficiency is likely more prevalent in IBD patients than healthy controls and correlates with worse clinical outcomes while normalization of 25(OH)D concentrations has been proposed to confer a reduced risk of hospitalization and surgery [7, 8]. Several cohort studies identified risk factors for vitamin D deficiency including diagnosis of CD *vs*. UC, disease activity, disease duration, gender, non-Caucasian ethnicity, smoking, body mass index [BMI] and concomitant medications, in particular therapy with TNF inhibitors [9–20]. However, many of these associations have been controversial between individual studies. In addition, it is obvious that seasonal variability and subsequent sun exposure will have a significant impact on patients’ vitamin D status [21]. Such seasonal variability with lower 25(OH)D concentrations in winter has been demonstrated in some studies [14–16, 22], but not in others [11, 17]. One study even observed a higher prevalence of vitamin D deficiency in summer as compared to winter [18]. Moreover, seasons may also directly affect IBD activity [23], which may in turn affect vitamin D status and vice versa. Most of the epidemiological studies on vitamin D status only accounted for season (summer was usually defined as the period between April and September while winter was usually defined as period between October and March)[12, 16], suggesting that a more detailed analysis of temporal influences of the seasons may be more appropriate to judge clinical risk factors for vitamin D deficiency. We therefore aimed to assess clinical risk factors for vitamin D deficiency after thoroughly controlling for seasonal variability with regard to the specific day of analysis in a retrospective analysis of outpatients treated at a tertiary referral center in Germany and analyzed the impact of vitamin D supplementation during follow-up.

## Materials and methods

### Study design

A retrospective chart review of 600 outpatient IBD patients treated at Freiburg University Hospital between January 2010 and December 2016 was performed. Patients with at least one serum 25(OH)D measurement (n = 384) were included. Patient characteristics, clinical and laboratory data were obtained from the computerized hospital information system and medical records. IBD-related parameters such as onset of disease, complications, surgery, Harvey Bradshaw Index [24], season of 25(OH)D measurement, previous and current vitamin D supplementation and medications were determined. The first 25(OH)D measurement recorded was defined as baseline time point. A subsequent 25(OH)D analysis within 24 months qualified as follow-up measurement (n = 208).

### Ethics approval and consent to participate

Retrospective analysis of patient charts was approved by the local ethics committee (University Hospital Freiburg, permit number 407/16). Consent of the included patients for this retrospective non-interventional analysis was waived by the ethics committee.

### Statistical analysis

Predictive factors for vitamin D deficiency (serum concentration < 20 ng/ml) were analyzed with univariable and multiple logistic regression models. The seasonal variation in serum 25(OH)D concentrations was modelled by sinusoidal regression into a sinus curve by day of the year of 25(OH)D analysis (x) using the equation *y* = *a*+c*sin(*x*+*b*) with a, b and c being the standardized regression coefficients. Two models were established using 86,554 patient serum samples analyzed at the central laboratory of our institution between March 2011 and June 2018 irrespective of the underlying diagnosis and vitamin D supplementation and the serum samples from the first time point of the retrospective cohort (n = 384) respectively. The models were then used to correct the 25(OH)D measurements from both time points of the study for seasonal variations by correcting for the difference between the annual average vitamin D concentration and the predicted vitamin D concentration of the specific day of analysis. Statistical analysis was performed using R (The R Foundation, https://www.r-project.org) and the Tidyverse packages as well as SPSS version 24 (IBM, Ehingen, Germany). P<0.05 was considered significant.

## Results

### High prevalence rates of vitamin D deficiency in IBD patients

384 consecutive patients with at least one documented analysis of their 25(OH)D serum concentration were identified by retrospective chart review. Of these, 68% (n = 256) suffered from Crohn’s disease, while ulcerative colitis (31%; n = 121) and indeterminate colitis were less common (2%; n = 7). Mean age was 43 years (range 18–85 years), 53% were female and mean body mass index amounted to 24 kg/m^2^ (range 14–42). Presence of concomitant extra-intestinal manifestations was recorded for 88/384 patients (23%). The majority of patients had previously suffered from IBD-related complications (n = 212; 55%) including intestinal stenosis, fistula, abscess, major bleeding, intestinal dysplasia, or a combination thereof. Previous IBD-related surgery had been performed in 44%. Current medical therapies included mesalazine (31%), topical (20%) or systemic corticosteroids (35%), thiopurines (29%), methotrexate (2%), TNF inhibitors (29%) and other therapies including vedolizumab, tacrolimus or cyclosporine A (4%). Vitamin D supplementation prior to the index visit was only documented in the charts of 57 patients (15%, mean dose 1400 iU/day). The majority of patients suffered from vitamin D deficiency (<20 ng/ml; n = 232; 60%) or insufficiency (20–30 ng/ml; n = 94; 25%), while only 58 patients (15%) had sufficient 25(OH)D serum concentrations (>30 ng/ml)[25]. There were no apparent differences in the prevalence of vitamin D deficiency between patients with CD and UC (deficiency, insufficiency and sufficiency for CD 63%, 24% and 13% and for UC 55%, 24% and 20%, respectively).

### Impact of seasonality on vitamin D status in IBD patients

**Fig 1:**
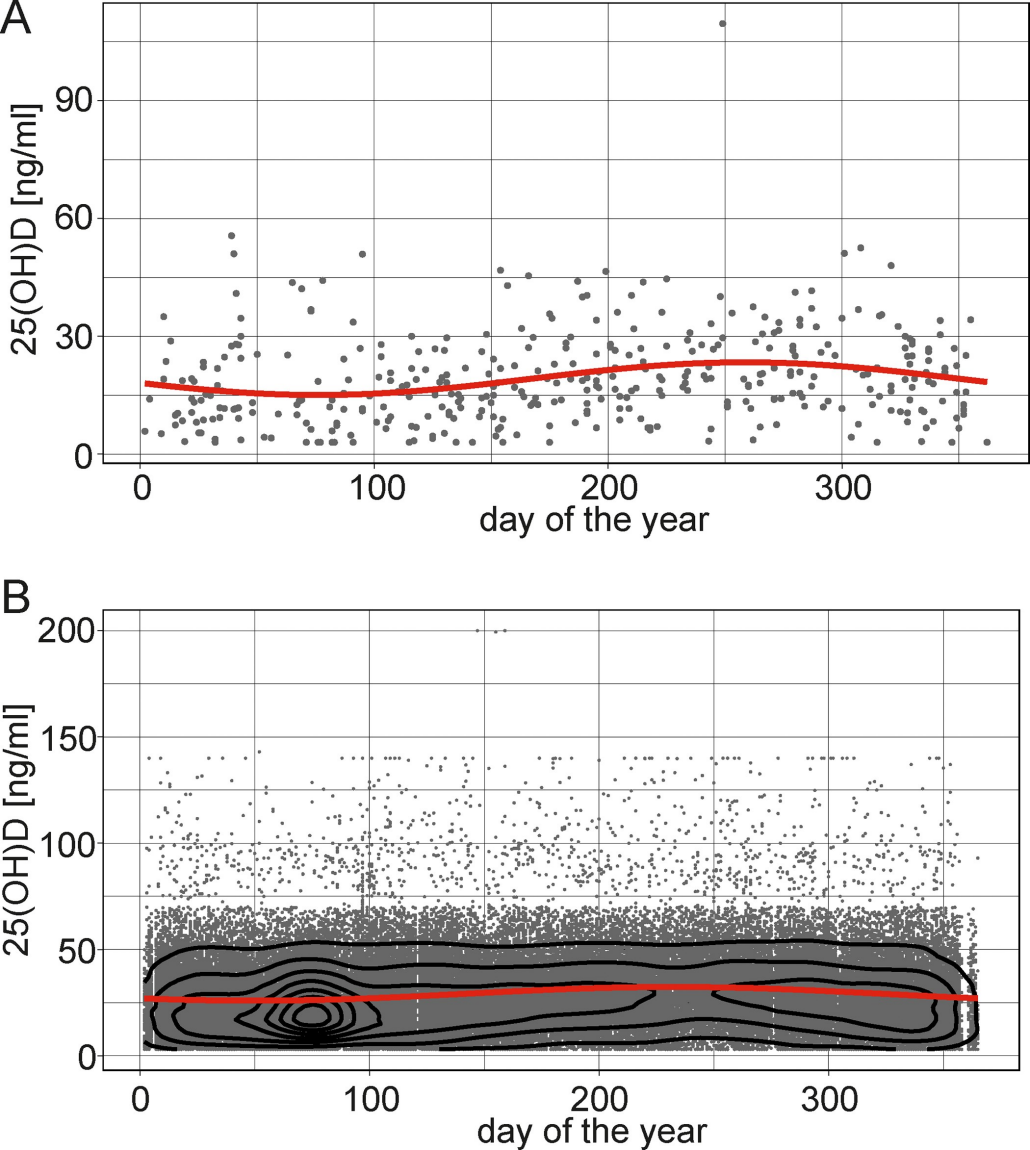
Sinusoidal pattern of 25(OH)D serum concentrations. (A) 25(OH)D serum concentrations measured in IBD patients at baseline and day of the year of the respective analysis are plotted. Sinusoidal regression analysis was performed to model a curve of the predicted 25(OH)D concentration over the year. (B) 25(OH)D serum concentrations measured in controls and day of the year of the respective analysis are plotted. Sinusoidal regression analysis was performed to model a curve of the predicted 25(OH)D concentration over the year. This fitted sinus curve had the equation *y* = 29.13+3.224*sin(*x*+0.861); F-statistic: 854.3 on 2 and 86548 DF, p-value: < 2.2e-16; MSE: 263.846.

To define clinical risk factors of vitamin D deficiency, univariable logistic regression analysis of several clinical parameters was performed and odds ratios (OR) with 95% confidence intervals (CI) were calculated. We assumed that vitamin D deficiency would have the greatest impact on potential disease complications and therefore compared deficient with non-deficient patients (i.e. all patients with 25(OH)D > 20 ng/ml) rather than distinguishing between vitamin D deficiency, insufficiency and sufficiency. This categorization has also been used in other studies [16, 18]. In a pooled analysis of patients with CD and UC, season of patient visit (winter/spring as compared to summer/fall; OR: 1.91; [95% CI: 1.13–3.28]; P = 0.02) was associated with a diagnosis of vitamin D deficiency. However, serum 25(OH)D concentrations displayed a distinctive seasonal variation over the course of the year (**Fig 1A**). Since seasonal variability may mask other clinical risk factors of vitamin D deficiency, a standard sinus curve was modeled by sinusoidal regression analysis using 86,554 patient serum samples analyzed at the Dept. of Clinical Chemistry of our institution between March 2011 and June 2018 irrespective of the underlying diagnosis and vitamin D supplementation. This analysis revealed a similar trend compared to that observed in IBD patients with lowest mean 25(OH)D concentrations observed on February 19^th^ (mean 26ng/ml) while the highest mean concentrations were observed on August 21^st^ (mean 32,4ng/ml) (**Fig 1B**).

To better control for seasonal variability, 25(OH)D serum concentrations of IBD patients were corrected for the difference between the annual average and the predicted 25(OH)D concentration of the specific day of analysis. The association between season of patient visit (winter/spring or summer/fall) and vitamin D deficiency was obviously no longer evident after normalization (OR 1.09; [95% CI: 0.65–1.84]; P = 0.75; normalized 25(OH)D serum concentrations are depicted in **S1 Fig**). After normalization, there was a significantly lower odds of patients with UC as compared to CD to suffer from vitamin D deficiency (UC *vs*. CD; OR 0.64; [95% CI: 0.41–0.99]; P = 0.047). Moreover, history of complications (fistula, stenosis, bleeding or dysplasia; OR 2.00; [95% CI: 1.3–3.0]; P = 0.001), previous IBD-related operations (OR 2.0; [95% CI: 1.3–3.1]; P = 0.001) and smoking (OR 2.1; [95% CI: 1.1–4.1]; P = 0.02) correlated positively while age at disease onset (OR 0.84; [95% CI: 0.71–0.99] per increasing 10 years of age; P = 0.04), initial disease manifestation during adulthood (>18 years of age; OR 0.51; [95% CI: 0.31–0.83]; P = 0.007) and ongoing vitamin D supplementation correlated negatively with vitamin D deficiency (OR 0.33; [95% CI: 0.18–0.59]; P<0.001). Moreover, patient-reported indicators of disease activity such as presence of diarrhea (defined as ≥4 liquid stools/24h; OR 2.8 [95% CI: 1.5–5.5]; P = 0.001), or general well-being defined as “bad” (OR 9.9; [95% CI: 2.7–64]; P = 0.003) correlated with the presence of vitamin D deficiency. Normalization for seasonal variability had only a modest impact on the associations between vitamin D deficiency and other clinical factors, suggesting that these clinical parameters were independent of the season of the year (**S1 Table**).

### Specific CD-related risk factors

**Fig 2:**
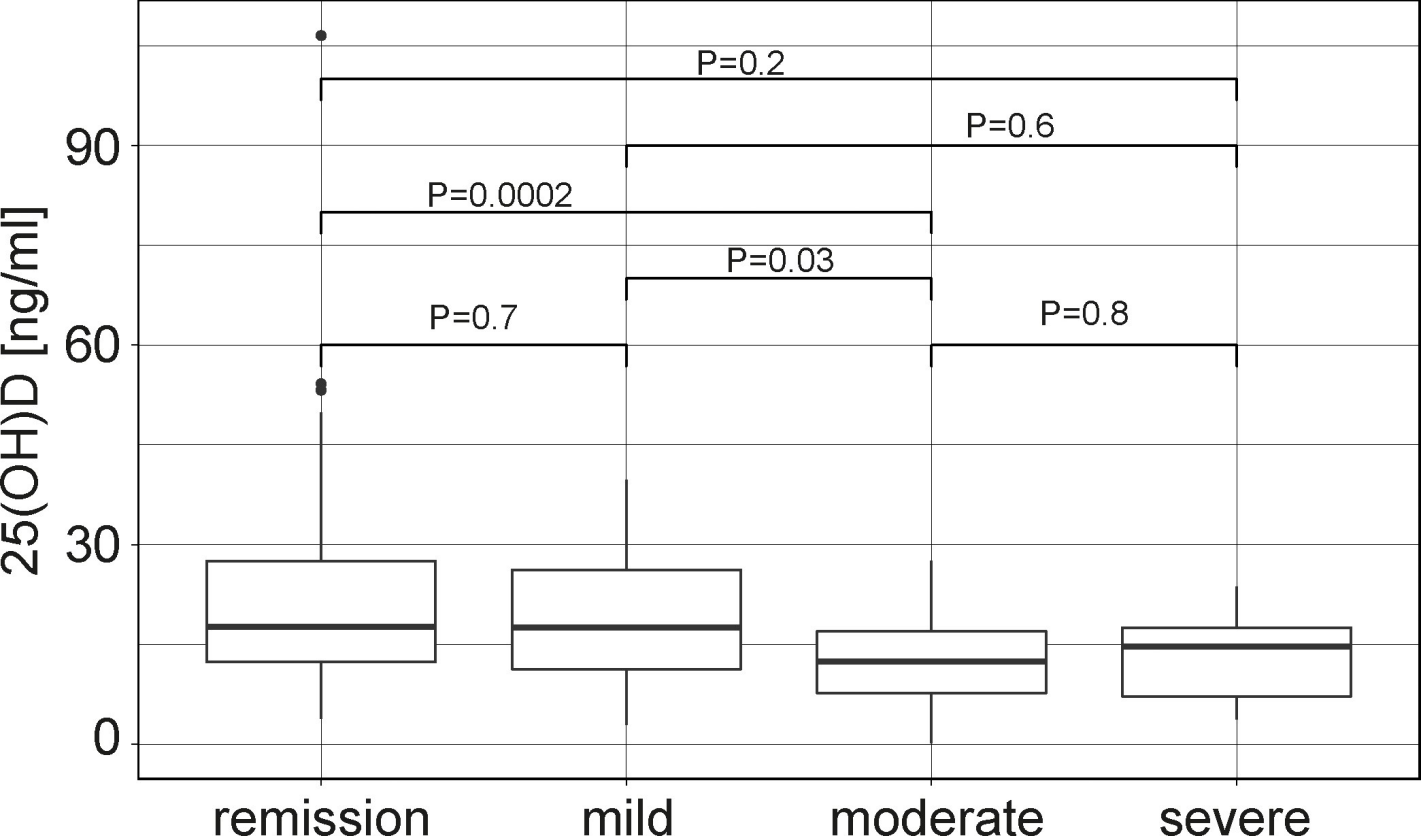
25(OH)D serum concentrations correlate with CD activity. Disease activity was determined using the HBI (categorized as remission, mild, moderate or severe disease). Differences between groups were assessed using Kruskal Wallis test and Dunn’s post test and P values are indicated.

Subgroup analysis of CD patients following normalization for seasonal variability revealed that moderate disease activity *vs*. remission as determined by Harvey Bradshaw Index (HBI, 8–16 points *vs*. <5 points; OR 9.1; [95% CI: 3.0–39.5]; P = 0.001) correlated with vitamin D deficiency, while there was no significant association with severe CD activity (HBI >16 points) *vs*. remission (OR 6; [95% CI: 1.0–114]; P = 0.1). Moreover, patients with moderate disease activity had significantly lower corrected absolute 25(OH)D concentrations as compared to patients in remission while the difference for severe activity did not reach statistical significance (**Fig 2**), which may be influenced by the fact that the number of patients with severe activity was rather limited in this outpatient cohort. Associations with smoking (OR 2.2; [95% CI: 1.1–4.8]; P = 0.03) and adult age at disease onset (OR 0.42; [95% CI: 0.22–0.77]; P = 0.006) were also confirmed in this subgroup. Concomitant medical therapies (e.g. corticosteroids, thiopurines or biologics including TNF inhibitors) and biomarkers of inflammation (concentrations of leukocytes, platelets or C-reactive protein) did not correlate with the diagnosis of vitamin D deficiency **(S2 Table)**. These findings indicate that the associations of disease activity, smoking and disease onset before adulthood with vitamin D deficiency in CD patients are indeed independent of seasonal variability.

**Fig 3:**
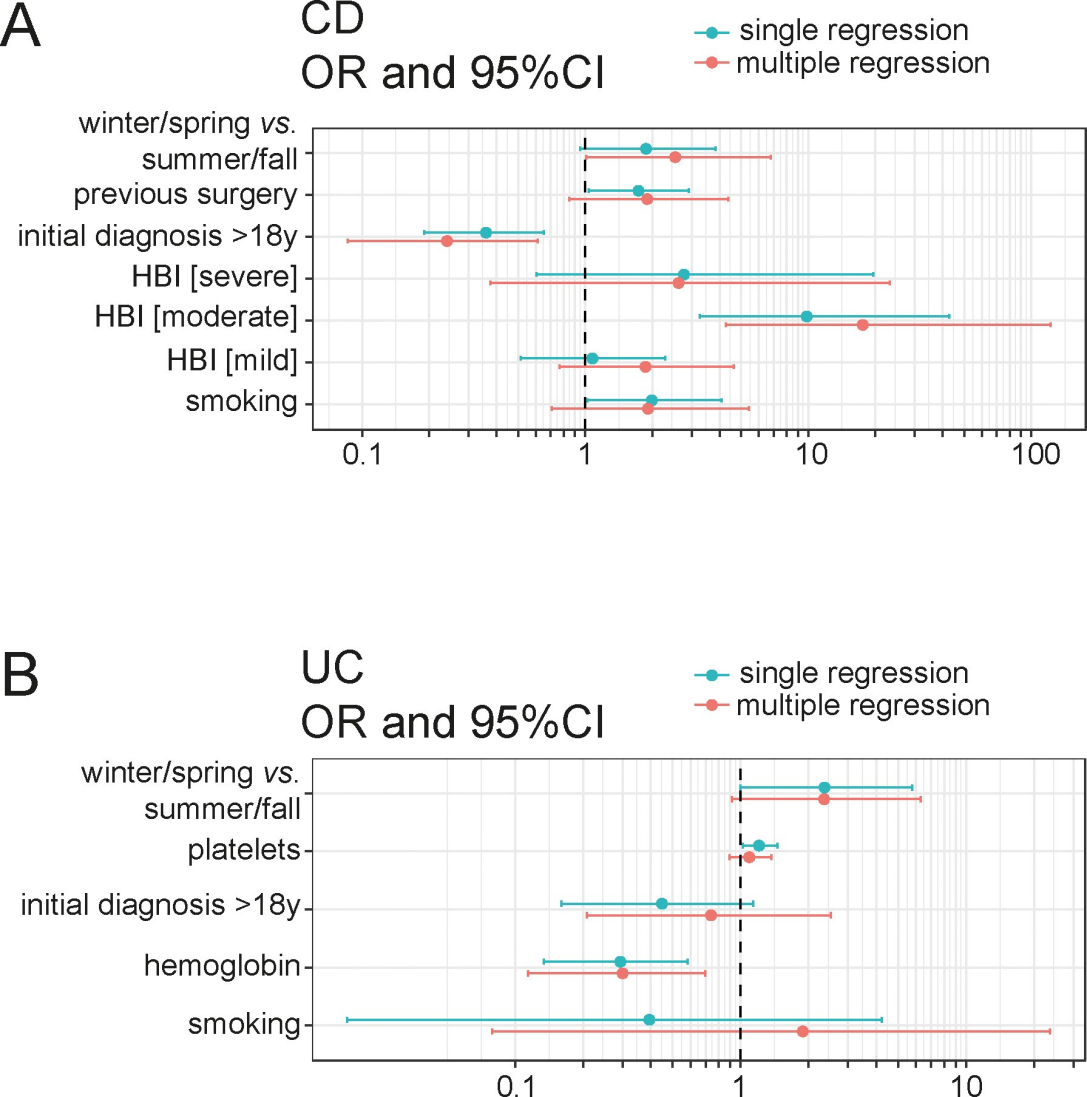
Univariable and multiple regression analyses to determine independent risk factors for vitamin D deficiency in patients with CD **(A)** and UC **(B)**. Models of multiple regression including the indicated clinical factors were used and are indicated as Forest plots.

To confirm these findings by another independent method, we performed a multiple regression analysis accounting for several parameters potentially contributing to the risk of vitamin D deficiency prior to normalization for seasonal variability. This model included smoking behavior, history of IBD-related operations, season of the year, disease activity categorized by HBI and age at disease onset (>18 *vs*. <18 years). In this multiple regression analysis, only primary diagnosis during adulthood correlated negatively (OR 0.24; [95% CI: 0.1–0.6]; P = 0.004) and HBI (moderate activity *vs*. remission OR 17.8; [95% CI: 4–122]; P = 0.0005) correlated positively with the presence of vitamin D deficiency while the season of patient visit was close to significance (winter/spring *vs*. summer/fall OR 2.5; [95% CI: 1.0–6.8]; P = 0.05) The associations between vitamin D deficiency and active smoking, history of CD-related operations, mild and severe disease activity did not reach statistical significance (**Fig 3A**).

### Specific UC-related risk factors

In UC patients, 25(OH)D analysis in winter/spring *vs*. summer/fall was marginally associated with vitamin D deficiency (OR 2.4; [95% CI: 1.0–5.8]; P = 0.05) and this correlation was obviously no longer evident after normalization for seasonal variability (OR 1.6; [95% CI: 0.7–3.8; P = 0.29). Univariable regression analysis after normalization revealed associations between vitamin D deficiency and platelet counts (OR 1.21; [95% CI: 1.02–1.46] per increasing 50,000/μl; P = 0.04), hemoglobin concentration (OR 0.32; [95% CI: 0.15–0.62] per increasing 3g/dl; P = 0.002) and hematocrit (OR 0.53; [95% CI: 0.33–0.81] per increasing 5%; P = 0.005), while no significant correlation was found for active smoking, patient reported disease activity, age at disease onset or history of extraintestinal manifestations (**S3 Table**).

Multiple regression analysis accounting for age at disease onset, active smoking, season of the year, hemoglobin and thrombocyte concentrations revealed that only hemoglobin concentrations correlated significantly with the presence of vitamin D deficiency (OR 0.3; [95% CI: 0.11–0.7]; P = 0.008; **Fig 3B**).

### Impact of vitamin D supplementation on disease outcomes

Patients with documented vitamin D deficiency had a higher probability to receive a prescription for vitamin D (OR 6.1; [95% CI: 3.9–9.7]; P<0.001). Follow-up measurements of 25(OH)D serum concentrations were available for 208 patients after a mean of 13 (±9) months. Among those, intake of vitamin D was documented for 96 patients (mean dose 1550 iU/day). While serum 25(OH)D concentrations did not change significantly in patients without documented supplementation (from 20±11 ng/ml to 18±11 ng/ml; P = 0.06 by Wilcoxon test), a significant increase was observed in patients with documented supplementation (from 17±12 ng/ml to 27±18 ng/ml; P<0.001 by Wilcoxon test). However, the majority of patients with vitamin D deficiency at baseline and documented supplementation remained deficient (31/68; 45%), while 25 patients (37%) improved to insufficiency and only 12 patients (18%) reached sufficient vitamin D concentrations.

To address the impact of seasonal variability on the course of vitamin D status, follow-up 25(OH)D concentrations were normalized by using the control sinusoidal regression analysis curve (depicted in Fig 1B). We focused the analysis of 25(OH)D concentrations on disease activity of CD patients given the rather limited numbers of patients with UC. CD activity was categorized as improvement within the predefined categories of the HBI (remission, mild, moderate or severe disease). Patients with improvement in HBI category had significantly increased 25(OH)D concentrations (Spearman’s correlation rho -0.28; P = 0.008), suggesting that clinical improvement indeed correlated with increasing 25(OH)D serum concentrations. In the next step, we performed a subgroup analysis comparing patients with an increase and decrease of their 25(OH)D concentrations by >10 ng/ml. We were unable to demonstrate differences in gender, age, diagnosis of CD *vs*. UC, age at disease onset, BMI, smoking or presence of extraintestinal manifestations. However, prescription of vitamin D by the treating physician (P<0.001) and documented vitamin D intake at follow-up (P<0.001) correlated with increased 25(OH)D concentrations, while disease activity at baseline (as determined by HBI for CD patients, P = 0.05), was close to significance. These findings suggest that vitamin D status is mainly influenced by patients’ compliance.

## Discussion

Vitamin D deficiency is highly prevalent in IBD patients, at least in northern and middle Europe, and affects up to 63% of patients [4, 16], which is consistent with the high prevalence of 60% reported in this retrospective cohort study. Several risk factors have been associated with a risk of vitamin D deficiency including age at diagnosis, season of the year, long disease duration, smoking, CD activity, female gender, non-Caucasian ethnicity and limited sunshine exposure [11, 13, 16, 18, 20–22]. Higher 25(OH)D concentrations correlate with decreased risk to develop IBD-related complications and better outcomes of TNF-inhibitor therapy [8, 15, 19]. We confirmed several of these established risk factors in our study (no vitamin D intake, disease onset during childhood and active smoking for UC and CD; disease activity and history of CD-related operations in patients with CD; anemia and thrombocytosis for UC). However, we found that 25(OH)D concentrations were indeed influenced by seasonal factors, most likely since vitamin D is mainly generated in the skin under control of UVB exposure. We therefore established a model to predict the 25(OH)D serum concentration for any given day of the year in both, patients with IBD from our retrospective cohort as well as an independent large local reference population using a sinusoidal regression analysis. A similar approach was used to predict seasonal and vitamin D-dependent variations of disease activity in patients with multiple sclerosis [26]. At least to our knowledge, this is the first systematic analysis of the impact of seasons as determined by day of the year on the vitamin D status of IBD patients. Our findings highlight the importance of considering seasonality when interpreting vitamin D lab results and optimizing patient care. Application of this novel approach to IBD patients is particularly useful for two reasons. First, we could confirm that the associations of most known clinical risk factors with vitamin D deficiency were indeed independent of seasonal variability and could be confirmed after normalization of 25(OH)D concentrations as well as by analysis of independent risk factors by multiple regression. Second, sinusoidal regression may be useful to better interpret the results of 25(OH)D testing in the clinic, since seasonal variations can be taken into account. Given the high financial burden of repeated 25(OH)D serum testing, our data suggest that 25(OH)D testing may be done preferably in late winter/early spring, since patients with clinically meaningful vitamin D deficiency may be reliably diagnosed at this time point. Moreover, physicians could adjust the 25(OH)D concentration with respect to the date of blood draw which would allow a better estimate of the respective vitamin D status.

Optimal 25(OH)D serum concentrations for IBD patients are still a matter of debate [4]. Current thresholds are derived from recommendations for prevention of osteoporosis and do not necessarily apply to IBD-related risk factors. In a prospective cohort, 25(OH)D concentrations < 20ng/ml have been associated with an increased risk to develop IBD-related complications [8], suggesting that it is prudent to keep 25(OH)D concentrations above this threshold or even better above 30ng/ml [27].

It is obvious that ongoing vitamin D supplementation protected against vitamin D deficiency. Moreover, follow-up analyses revealed that correction of vitamin D deficiency mainly depends on patients’ compliance as well as vitamin D dosage. However, our data indicate that only a minority of deficient patients achieve a normalization of their 25(OH)D concentrations. Therefore, there is a clear need for better and repeated measures of patient education. Several findings indicate that vitamin D has anti-inflammatory effects on the immune system [4, 28] and it was shown to inhibit pro-inflammatory TH1 and TH17 cytokine production in CD patients *in vivo* [29]. The question whether vitamin D deficiency is cause or consequence of disease activity remains enigmatic. Moreover, clinical trials addressing the impact of vitamin D supplementation on IBD disease outcomes have yielded conflicting results [30–33], which may also be affected by implementing a suboptimal trial design [34]. Our finding that CD patients with improved disease activity at follow-up also had significantly increased 25(OH)D concentrations is in favor of the hypothesis that vitamin D indeed exerts protective and anti-inflammatory effects. The impact of vitamin D on immune cell function as well as therapeutic application of vitamin D to affect clinically meaningful IBD outcomes should therefore be addressed in prospective trials.

### Conclusions

25(OH)D concentrations of IBD patients are strongly influenced by seasonal factors, which can be predicted for every day of the year by a sinus curve-based algorithm. Our data indicate that the risk to develop vitamin D deficiency is mainly determined by these seasonal factors, patient compliance as well as disease activity. Moreover, improved disease activity of CD patients over time was associated with increasing serum 25(OH)D concentrations. These findings suggest that vitamin D supplementation should be performed in IBD patients, particularly during winter/spring seasons and in those with active disease.

## Supporting information

S1 Fig Impact of normalization for seasonality of serum 25(OH)D concentrations in IBD patients. (TIF)

S1 Table Univariable regression analysis of associations between clinical parameters of pooled CD and UC patients with vitamin D deficiency. (DOCX)

S2 Table Univariable regression analysis of associations between clinical parameters of CD patients with vitamin D deficiency. (DOCX)

S3 Table Univariable regression analysis of associations between clinical parameters of UC patients with vitamin D deficiency. (DOCX)

## Abbreviations

CD: Crohn’s disease
UC: ulcerative colitis
IBD: inflammatory bowel disease
OR: odds ratio
CI: confidence interval
25(OH)D: 25-(OH)-vitamin D
HBI: Harvey Bradshaw Index
