# Supplementary figures and images for: Seasonal variability of vitamin D status in patients with inflammatory bowel disease – A retrospective cohort study
Source: PLoS One. 2019 May 23;14(5):e0217238. doi: 10.1371/journal.pone.0217238 (PMC6532907; doi:10.1371/journal.pone.0217238)

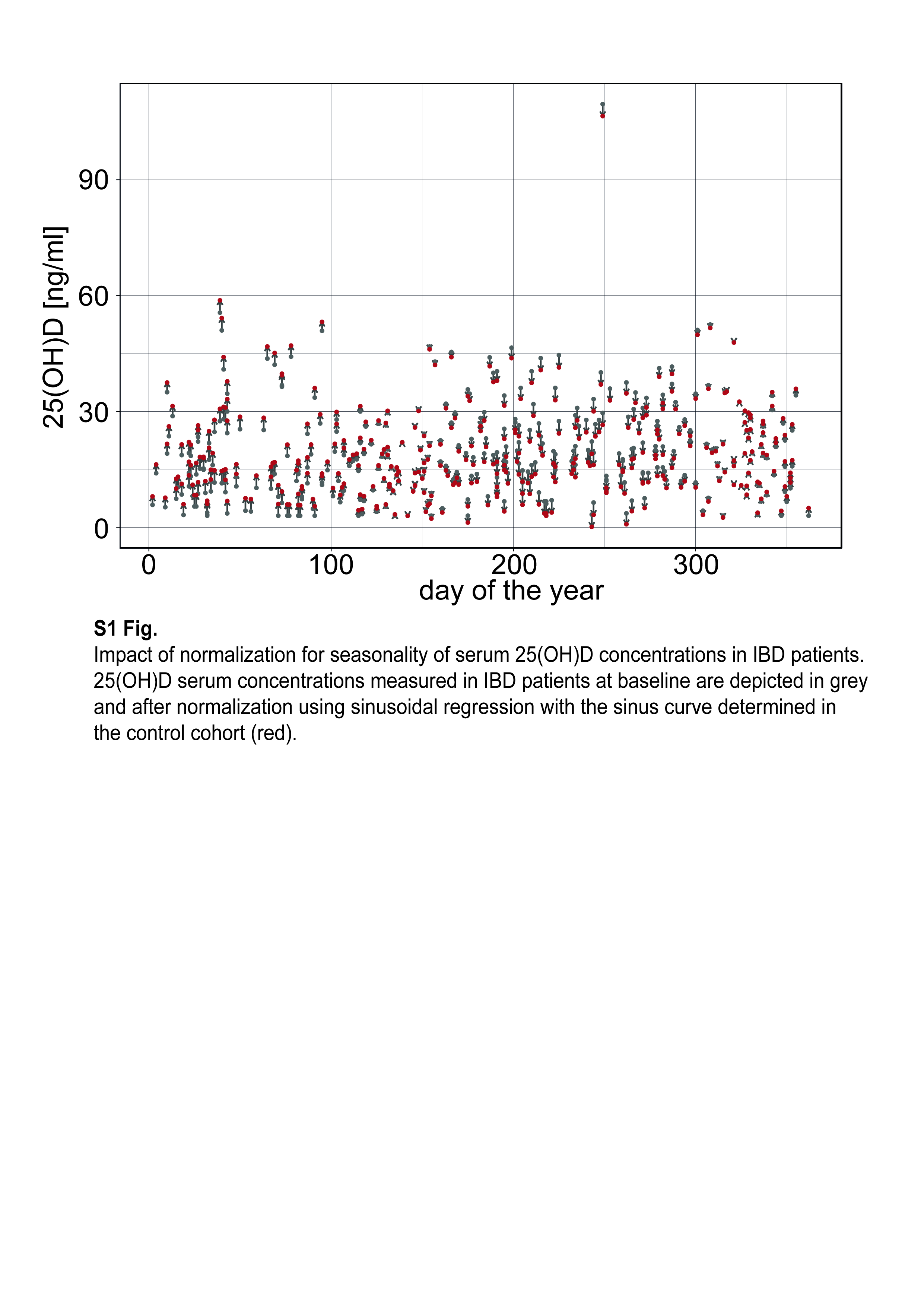

Supplement: S1 Fig — (TIF) [file pone.0217238.s001.tif]
