# Supplementary material for: Seasonal variability of vitamin D status in patients with inflammatory bowel disease – A retrospective cohort study
Source: PLoS One. 2019 May 23;14(5):e0217238. doi: 10.1371/journal.pone.0217238 (PMC6532907; doi:10.1371/journal.pone.0217238)
Supplement: S1 Table — (DOCX) [file pone.0217238.s002.docx]

**S1 Table**: Univariable regression analysis of associations between clinical parameters of pooled CD and UC patients with vitamin D deficiency.

| **Pooled UC and CD** | | **Univariable regression with normalized 25(OH)D concentrations** | | | **Univariable regression with non-normalized 25(OH)D concentrations** | | |
| --- | --- | --- | --- | --- | --- | --- | --- |
| **Parameter** | **comparator** | **OR** | **95% CI** | **P value** | **OR** | **95% CI** | **P value** |
| Age | per increasing 10 years | 0.96 | 0.84 - 1.1 | 0.54 | 0.89 | 0.77 - 1.02 | 0.08 |
| Gender | male *vs*.f  female | 1.42 | 0.94 - 2.15 | 0.10 | 1.25 | 0.83 - 1.89 | 0.28 |
| Diagnosis | UC *vs*.CD | 0.64 | 0.41 - 0.99 | **0.047** | 0.74 | 0.48 - 1.16 | 0.19 |
| Age at initial diagnosis | per increasing 10 years | 0.84 | 0.71 - 0.99 | **0.04** | 0.79 | 0.67 - 0.93 | **0.006** |
| Age at initial diagosis | during *vs*.prior , adulthood | 0.51 | 0.31 - 0.83 | **0.007** | 0.43 | 0.26 - 0.69 | **0.001** |
| BMI | per increasing 5 kg/m2 | 0.9 | 0.71 - 1.13 | 0.37 | 0.90 | 0.71 - 1.13 | 0.35 |
| Smoking | active *vs*.non-smoking | 2.01 | 1.13 - 4.12 | **0.02** | 2.01 | 1.09 - 3.88 | **0.03** |
| Extraintestinal manifestations | *vs*.none | 1.07 | 0.66 - 1.77 | 0.78 | 0.85 | 0.52 - 1.38 | 0.50 |
| Previous IBD related surgery | *vs*.none | 2.0 | 1.31 - 3.07 | **0.001** | 1.74 | 1.15 - 2.65 | **0.01** |
| Previous IBD related complications | *vs*. none | 1.99 | 1.31 - 3.03 | **0.001** | 1.64 | 1.08 - 2.48 | **0.02** |
| General well-being: reduced | *vs*. normal | 1.70 | 0.93 - 3.17 | 0.09 | 1.35 | 0.75 - 2.47 | 0.32 |
| General well-being: bad | *vs*. normal | 9.9 | 2.73 - 6.16 | **0.003** | 6.6 | 2.10 - 29.2 | **0.004** |
| Stool frequency | per increasing liquid bowel movement | 1.18 | 1.08 - 1.32 | **0.001** | 1.14 | 1.05 - 1.26 | **0.006** |
| Abdominal pain | mild *vs*. none | 1.06 | 0.54 - 2.13 | 0.86 | 0.76 | 0.38 - 1.49 | 0.42 |
| Abdominal pain | moderate *vs*. none | 2.19 | 0.90 - 5.90 | 0.1 | 2.19 | 0.90 - 5.90 | 0.10 |
| Abdominal pain | severe *vs*. none | 3.13 | 0.77 - 21.13 | 0.16 | 1.86 | 0.51 - 8.80 | 0.38 |
| Season of the year | winter/spring *vs*. summer/fall | 1.09 | 0.65 - 1.84 | 0.75 | 1.91 | 1.13 - 3.28 | **0.02** |
| Vitamin D substitution | *vs*. none | 0.33 | 0.18 - 0.59 | **<0.001** | 0.30 | 0.16 - 0.53 | **<0.001** |
| C-reactive protein | per increasing 10 mg/dl | 1.09 | 0.98 - 1.23 | 0.14 | 1.07 | 0.97 - 1.21 | 0.20 |
| Leucocytes | per increasing 5000/µl | 1.09 | 0.80 - 1.50 | 0.59 | 1.01 | 0.74 - 1.38 | 0.95 |
| Thrombocytes | per increasing 50,000/µl | 1.12 | 1.01 - 1.24 | **0.03** | 1.15 | 1.04 - 1.28 | **0.008** |
| Hemoglobin | per increasing 3 g/dl | 0.62 | 0.42 - 0.89 | **0.01** | 0.55 | 0.37 - 0.79 | **0.002** |
| Hematocrit | per increasing 5% | 0.78 | 0.61 - 0.98 | **0.04** | 0.71 | 0.55 - 0.89 | **0.005** |
| Albumin | per increasing g/dl | 0.84 | 0.56 - 1.26 | 0.40 | 0.91 | 0.61 - 1.36 | 0.64 |
| Ferritin | per increasing 50 ng/ml | 0.98 | 0.91 - 1.06 | 0.64 | 0.99 | 0.91 - 1.06 | 0.68 |
| Vitamin,B12 | per increasing 100 pg/ml | 0.97 | 0.91 - 1.03 | 0.27 | 0.98 | 0.92 - 1.04 | 0.53 |
| Calprotectin | per increasing 100 mg/kg | 0.86 | 0.68 - 1.04 | 0.13 | 0.90 | 0.73 - 1.10 | 0.30 |
| Topical steroids | active *vs*. no therapy | 1.66 | 1.03 - 2.68 | **0.04** | 1.25 | 0.78 - 2.02 | 0.35 |
| Topical steroids | previous *vs*. no therapy | 2.17 | 1.19 - 4.03 | **0.01** | 1.77 | 0.98 - 3.27 | 0.06 |
| Prednisone | active *vs*. no therapy | 1.31 | 0.66 - 2.57 | 0.43 | 1.01 | 0.5 - 1.99 | 0.98 |
| Prednisone | previous *vs.* no therapy | 1.05 | 0.51 - 2.1 | 0.90 | 0.81 | 0.39 - 1.63 | 0.55 |
| Azathioprine | active *vs*. no therapy | 0.76 | 0.46 - 1.24 | 0.27 | 0.78 | 0.48 - 1.28 | 0.33 |
| Azathioprine | previous *vs*. no therapy | 0.81 | 0.48 - 1.38 | 0.45 | 0.87 | 0.51 - 1.47 | 0.60 |
| Methotrexate | active *vs*. no therapy | 0.87 | 0.41 - 1.87 | 0.71 | 1.04 | 0.50 - 2.28 | 0.91 |
| Methotrexate | previous *vs*. no therapy | 0.31 | 0.043 - 1.63 | 0.18 | 0.33 | 0.05 - 1.71 | 0.20 |
| 6-Mercaptopurine | active *vs*. no therapy | 0.70 | 0.25 - 2.05 | 0.51 | 1.32 | 0.46 - 4.33 | 0.62 |
| 6-Mercaptopurine | previous *vs*. no therapy | 0.21 | 0.03 - 0.91 | 0.06 | 0.40 | 0.08 - 1.64 | 0.21 |
| 5-ASA | active *vs*. no therapy | 1.24 | 0.72 - 2.17 | 0.44 | 1.31 | 0.77 - 2.28 | 0.33 |
| 5-ASA | previous *vs*. no therapy | 0.58 | 0.36 - 0.94 | **0.03** | 0.69 | 0.43 - 1.11 | 0.13 |
| TNF inhibitors | active *vs*. no therapy | 1.28 | 0.66 - 2.60 | 0.47 | 1.23 | 0.63 - 2.46 | 0.55 |
| TNF inhibitors | previous *vs*. no therapy | 1.12 | 0.70 - 1.79 | 0.64 | 1.23 | 0.77 - 1.97 | 0.38 |
| Diarrhea | ≥4 liquid stools *vs*. none | 2.84 | 1.52 - 5.50 | **0.001** | 2.40 | 1.30 - 4.53 | **0.006** |
