# Supplementary material for: Seasonal variability of vitamin D status in patients with inflammatory bowel disease – A retrospective cohort study
Source: PLoS One. 2019 May 23;14(5):e0217238. doi: 10.1371/journal.pone.0217238 (PMC6532907; doi:10.1371/journal.pone.0217238)
Supplement: S2 Table — (DOCX) [file pone.0217238.s003.docx]

**S2 Table:** Univariable regression analysis of associations between clinical parameters of CD patients with vitamin D deficiency

| **CD** | | **Univariable regression with normalized 25(OH)D concentrations** | | | **Univariable regression with non-normalized 25(OH)D concentrations** | | |
| --- | --- | --- | --- | --- | --- | --- | --- |
| **Parameter** | **comparator** | **OR** | **95% CI** | **P value** | **OR** | **95% CI** | **P value** |
| Age | per increasing 10 years | 0.97 | 0.81 - 1.15 | 0.69 | 0.91 | 0.77 - 1.08 | 0.30 |
| Gender | male *vs*, female | 1.26 | 0.75 - 2.13 | 0.39 | 1.03 | 0.62 - 1.71 | 0.92 |
| Age at initial diagnosis | per increasing 10 years | 0.84 | 0.68 - 1.03 | 0.1 | 0.78 | 0.63 - 0.96 | **0.02** |
| Age at initial diagosis | during *vs*. prior , adulthood | 0.42 | 0.22 - 0.77 | **0.006** | 0.36 | 0.19 - 0.65 | **0.001** |
| BMI | per increasing 5 kg/m2 | 0.87 | 0.65 - 1.16 | 0.33 | 0.81 | 0.60 - 1.08 | 0.15 |
| Smoking | active *vs*. non-smoking | 2.23 | 1.12 - 4.81 | **0.03** | 1.99 | 1.02- 4.09 | 0.05 |
| Extraintestinal manifestations | *vs*. none | 0.83 | 0.45 - 1.55 | 0.55 | 0.79 | 0.43 - 1.47 | 0.46 |
| Previous IBD related surgery | *vs*. none | 1.62 | 0.96 - 2.74 | 0.07 | 1.73 | 1.04 - 2.91 | **0.04** |
| Previous IBD related complications | *vs*. none | 1.83 | 1.02 - 3.26 | **0.04** | 1.68 | 0.95 - 3.00 | 0.08 |
| General well-being: reduced | *vs*. normal | 1.67 | 0.91 - 3.11 | 0.10 | 1.38 | 0.76 - 2.53 | 0.30 |
| General well-being: bad | *vs*. normal | 18.5 | 3.6- 339 | **0.005** | 9.6 | 2.61 - 62.2 | **0.003** |
| Stool frequency | per increasing liquid bowel movement | 1.18 | 1.07 - 1.32 | **0.002** | 1.14 | 1.05 - 1.27 | **0.007** |
| Abdominal pain | mild *vs*. none | 1.05 | 0.53 - 2.14 | 0.89 | 0.77 | 0.38 - 1.53 | 0.45 |
| Abdominal pain | moderate *vs*. none | 2.13 | 0.87 - 5.75 | 0.11 | 2.21 | 0.91 - 5.96 | 0.10 |
| Abdominal pain | severe *vs*. none | 3.04 | 0.74 - 20.57 | 0.17 | 1.87 | 0.51 - 8.88 | 0.38 |
| HBI | mild activity *vs*. remission | 1.36 | 0.65 - 2.95 | 0.42 | 1.08 | 0.51 - 2.28 | 0.85 |
| HBI | moderate activity *vs*. remission | 9 | 3 - 40 | **0.001** | 10 | 3 - 43 | **<0.001** |
| HBI | severe activity *vs*. remission | 6 | 1.0 - 114 | 0.10 | 2.77 | 0.61 - 19.53 | 0.23 |
| Season of the year | winter/spring *vs*. summer/fall | 0.94 | 0.48 - 1.85 | 0.85 | 1.87 | 0.95 - 3.84 | 0.08 |
| Vitamin D substitution | *vs*. none | 0.32 | 0.16 - 0.65 | **0.002** | 0.29 | 0.14 - 0.58 | **0.001** |
| C-reactive protein | per increasing 10 mg/dl | 1.03 | 0.92 - 1.18 | 0.60 | 1.04 | 0.92 - 1.18 | 0.58 |
| Leucocytes | per increasing 5000/µl | 0.95 | 0.62 - 1.45 | 0.80 | 0.86 | 0.57 - 1.30 | 0.46 |
| Thrombocytes | per increasing 50000/µl | 1.08 | 0.95 - 1.23 | 0.27 | 1.13 | 1.00 - 1.30 | 0.07 |
| Hemoglobin | per increasing 3 g/dl | 0.80 | 0.50 - 1.24 | 0.33 | 0.65 | 0.40 - 1.01 | 0.07 |
| Hematocrit | per increasing 5% | 0.90 | 0.67 - 1.19 | 0.47 | 0.78 | 0.57 - 1.04 | 0.10 |
| Albumin | per increasing g/dl | 0.82 | 0.51 - 1.29 | 0.38 | 0.93 | 0.59 - 1.47 | 0.75 |
| Ferritin | per increasing 50 ng/ml | 0.99 | 0.92 - 1.09 | 0.85 | 0.99 | 0.91 - 1.08 | 0.83 |
| Vitamin,B12 | per increasing 100 pg/ml | 0.84 | 0.71 - 0.96 | **0.03** | 0.89 | 0.77 - 0.99 | 0.09 |
| Calprotectin | per increasing 100 mg/kg | 0.67 | 0.41 - 0.92 | **0.045** | 0.79 | 0.55 - 1.01 | 0.10 |
| Topical steroids | active *vs*. no therapy | 1.78 | 0.97 - 3.29 | 0.06 | 1.43 | 0.78 - 2.61 | 0.25 |
| Topical steroids | previous *vs*. no therapy | 1.76 | 0.86 - 3.66 | 0.13 | 1.63 | 0.80 - 3.37 | 0.18 |
| Prednisone | active *vs*. no therapy | 1.93 | 0.72 - 5.11 | 0.18 | 2.18 | 0.83 - 5.82 | 0.11 |
| Prednisone | previous *vs*. no therapy | 1.48 | 0.53 - 4.07 | 0.44 | 1.57 | 0.57 - 4.35 | 0.38 |
| Azathioprine | active *vs*. no therapy | 0.59 | 0.31 - 1.10 | 0.10 | 0.71 | 0.38 - 1.30 | 0.26 |
| Azathioprine | previous *vs*. no therapy | 0.75 | 0.38 - 1.48 | 0.40 | 0.83 | 0.43 - 1.61 | 0.58 |
| Methotrexate | active *vs*. no therapy | 0.77 | 0.33 - 1.85 | 0.55 | 1.31 | 0.56 - 3.33 | 0.55 |
| Methotrexate | previous *vs*. no therapy | 0.17 | 0.01 - 1.36 | 0.13 | 0.21 | 0.01 - 1.63 | 0.17 |
| 6-Mercaptopurine | active *vs*. no therapy | 0.51 | 0.16 - 1.68 | 0.26 | 1.23 | 0.38 - 4.72 | 0.74 |
| 6-Mercaptopurine | previous *vs*. no therapy | 0.17 | 0.01 - 1.35 | 0.13 | 0.62 | 0.07 - 5.20 | 0.63 |
| 5-ASA | active *vs*. no therapy | 1.41 | 0.73 - 2.82 | 0.31 | 1.46 | 0.76 - 2.88 | 0.26 |
| 5-ASA | previous *vs*. no therapy | 0.89 | 0.46 - 1.76 | 0.74 | 0.85 | 0.44 - 1.65 | 0.62 |
| TNF inhibitors | active *vs*. no therapy | 0.76 | 0.33 - 1.81 | 0.52 | 0.79 | 0.34 - 1.84 | 0.58 |
| TNF inhibitors | previous *vs*. no therapy | 0.97 | 0.55 - 1.73 | 0.92 | 1.18 | 0.70 - 2.09 | 0.58 |
| Diarrhea | ≥4 liquid stools *vs*. none | 2.80 | 1.48 - 5.50 | **0.002** | 2.43 | 1.31 - 4.63 | **0.006** |
