# Supplementary material for: Seasonal variability of vitamin D status in patients with inflammatory bowel disease – A retrospective cohort study
Source: PLoS One. 2019 May 23;14(5):e0217238. doi: 10.1371/journal.pone.0217238 (PMC6532907; doi:10.1371/journal.pone.0217238)
Supplement: S3 Table — (DOCX) [file pone.0217238.s004.docx]

**S3 Table:** Univariable regression analysis of associations between clinical parameters of UC patients with vitamin D deficiency

| **UC** | | **Univariable regression with normalized 25(OH)D concentrations** | | | **Univariable regression with non-normalized 25(OH)D concentrations** | | |
| --- | --- | --- | --- | --- | --- | --- | --- |
| **Parameter** | **comparator** | **OR** | **95% CI** | **P value** | **OR** | **95% CI** | **P value** |
| Age | per increasing 10 years | 0.90 | 0.71 - 1.13 | 0.37 | 0.81 | 0.64 - 1.02 | 0.08 |
| Gender | male *vs*, Female | 1.73 | 0.84 - 3.60 | 0.14 | 1.62 | 0.79 - 3.35 | 0.19 |
| Age at initial diagnosis | per increasing 10 years | 0.81 | 0.6 - 1.08 | 0.15 | 0.79 | 0.58 - 1.05 | 0.11 |
| Age at initial diagosis | during *vs*. prior adulthood | 0.65 | 0.25 - 1.61 | 0.36 | 0.45 | 0.16 - 1.14 | 0.10 |
| BMI | per increasing 5 kg/m2 | 0.93 | 0.61 - 1.40 | 0.71 | 1.04 | 0.69 - 1.59 | 0.86 |
| Smoking | active *vs*. non-smoking | 0.41 | 0.02 - 4.37 | 0.47 | 0.40 | 0.02 - 4.22 | 0.45 |
| Extraintestinal manifestations | *vs*. none | 1.87 | 0.82 - 4.44 | 0.15 | 1.05 | 0.47 - 2.40 | 0.91 |
| Previous IBD related surgery | *vs*. none | 2.83 | 0.92 - 10.65 | 0.09 | 1.40 | 0.49 - 4.39 | 0.54 |
| Previous IBD related complications | *vs*. none | 2.02 | 0.78 - 5.68 | 0.16 | 1.52 | 0.60 - 4.11 | 0.39 |
| Season of the year | winter/spring *vs*. summer/fall | 1.59 | 0.68 - 3.82 | 0.29 | 2.36 | 1.00- 5.76 | 0.05 |
| Vitamin D substitution | *vs*. none | 0.32 | 0.10 - 0.87 | **0.03** | 0.30 | 0.1 - 0.83 | **0.03** |
| C-reactive protein | per increasing 10 mg/dl | 1.19 | 0.96 - 1.67 | 0.17 | 1.17 | 0.94 - 1.62 | 0.23 |
| Leucocytes | per increasing 5000/µl | 1.45 | 0.87 - 2.52 | 0.17 | 1.43 | 0.86 - 2.49 | 0.18 |
| Thrombocytes | per increasing 50,000/µl | 1.21 | 1.02 - 1.46 | **0.04** | 1.21 | 1.02 - 1.46 | **0.04** |
| Hemoglobin | per increasing 3 g/dl | 0.32 | 0.15 - 0.62 | **0.002** | 0.29 | 0.13 - 0.58 | **0.001** |
| Hematocrit | per increasing 5% | 0.53 | 0.33 - 0.81 | **0.005** | 0.49 | 0.30 - 0.76 | **0.003** |
| Albumin | per increasing g/dl | 0.99 | 0.39 - 2.55 | 0.98 | 0.87 | 0.34 - 2.14 | 0.76 |
| Ferritin | per increasing 50 ng/ml | 0.92 | 0.74 - 1.10 | 0.37 | 0.93 | 0.75 - 1.12 | 0.44 |
| Vitamin,B12 | per increasing 100 pg/ml | 1.05 | 0.96 - 1.23 | 0.36 | 1.07 | 0.98 - 1.30 | 0.28 |
| Topical steroids | per increasing 100 mg/kg | 1.67 | 0.75 - 3.76 | 0.21 | 1.06 | 0.47 - 2.35 | 0.89 |
| Topical steroids | previous *vs*. no therapy | 3.07 | 0.95 - 11.14 | 0.07 | 2.07 | 0.64 - 7.50 | 0.24 |
| Prednisone | active *vs*. no therapy | 0.69 | 0.24 - 1.98 | 0.50 | 0.45 | 0.14 - 1.31 | 0.15 |
| Prednisone | previous *vs*. no therapy | 0.82 | 0.28 - 2.34 | 0.71 | 0.57 | 0.18 - 1.68 | 0.32 |
| Azathioprine | active *vs*. no therapy | 0.86 | 0.37 - 2.04 | 0.74 | 0.79 | 0.33 - 1.87 | 0.59 |
| Azathioprine | previous *vs*. no therapy | 0.74 | 0.30 - 1.83 | 0.52 | 0.87 | 0.35 - 2.18 | 0.77 |
| Methotrexate | active *vs*. no therapy | 0.83 | 0.15 - 4.67 | 0.83 | 0.36 | 0.05 - 1.92 | 0.25 |
| Methotrexate | previous *vs*. no therapy | 0.83 | 0.03 - 21 | 0.90 | 0.72 | 0.03 - 18.49 | 0.82 |
| 6-Mercaptopurine | active *vs*. no therapy | 1.64 | 0.153 - 36 | 0.69 | 1.47 | 0.14 - 32.15 | 0.76 |
| 6-Mercaptopurine | previous *vs*. no therapy | 0.27 | 0.01 - 2.21 | 0.27 | 0.255 | 0.01 - 1.98 | 0.23 |
| 5-ASA | active *vs*. no therapy | 0.88 | 0.25 - 2.92 | 0.84 | 0.77 | 0.22 - 2.52 | 0.67 |
| 5-ASA | previous *vs*. no therapy | 0.37 | 0.12 - 1.07 | 0.08 | 0.48 | 0.15 - 1.36 | 0.18 |
| TNF inhibitors | active *vs*. no therapy | 2.75 | 0.85 - 10.64 | 0.11 | 2.47 | 0.77 - 9.55 | 0.15 |
| TNF inhibitors | previous *vs*. no therapy | 1.23 | 0.52 - 2.95 | 0.64 | 1.27 | 0.54 - 3.08 | 0.59 |

,,
